# Cervical HPV infection and related diseases among 149,559 women in Fujian: an epidemiological study from 2018 to 2023

**DOI:** 10.3389/fmicb.2024.1418218

**Published:** 2024-06-19

**Authors:** Kun Lin, Qiyang Hong, Ya Fu, Haijian Tu, Hua Lin, Jiexiang Huang, Yajing Hu, Minjun Huang, Mingqiao Chen

**Affiliations:** ^1^Prenatal Diagnosis Center, The Affiliated Hospital of Putian University, Putian University, Putian, China; ^2^Department of Laboratory Medicine, The First Affiliated Hospital, Fujian Medical University, Fuzhou, China; ^3^Institute of Basic Medical Sciences, Chinese Academy of Medical Sciences and Peking Union Medical College, Beijing, China; ^4^Department of Laboratory Medicine, The Affiliated Hospital of Putian University, Putian University, Putian, China; ^5^Department of Women Health Care, Putian Maternity and Child Health Care Hospital, Putian, China

**Keywords:** human papillomavirus, epidemiological features, cervical pathology, age, female

## Abstract

**Objectives:**

To comprehensively analyze the epidemiological features of human papillomavirus (HPV) and HPV-related cervical diseases in females aged 35–64 years.

**Methods:**

A total of 149,559 samples of exfoliated cervical cells screened for HPV and related cervical lesions from January 2018 to December 2023 were enrolled. The prevalence of 15 high-risk and 6 low-risk HPV genotypes were detected, and the cervical cytology were analyzed. The impact of single and multiple HPV infections was characterized, and the effect of age was studied.

**Results:**

The cervix cytology was normal in 86.60% of the females, while 7.13% of the females were diagnosed with cervix inflammation, 0.60% with ASC-US, 0.22% with ASC-H, 0.72% with LSIL, 0.49% with HSIL, 0.03% with ICC. The highest median age was observed in ASC-H group with 54 years old. Females with primary school education or lower have the highest positive rates. The overall HPV prevalence was 8.60%. The relatively prevalent HPV types were HPV52, 58, 16, 39, 51. HPV16, HPV18, HPV58, HPV33 and HPV52 were the top5 predominant types in ICC patients. 17.41% females suffered from multiple HPV infection with the most frequently co-infection subtypes being HPV52, HPV58 and HPV16. The prevalence of all HPV subtypes increased with age. Multiple HPV infections accounted for a larger proportion in those aged above 55 years. The peak HPV16 prevalence was observed in ICC group in cases aged 45–49 and 55–59. The peak HPV33 prevalence was observed in younger individuals aged 40–44 who developed ICC.

**Conclusion:**

More action should be taken against HPV33 infection.

## 1 Introduction

Cervical cancer (CC) ranks in the top three cancers in women of all ages, with an estimated 604,000 new cases and 342,000 deaths worldwide in 2020. Approximately, 90% of the new cases and deaths occurred in low- and middle-income countries (Sung et al., 2021). China and India together contributed to more than one-third of the global CC burden, with 106,000 cases and 48,000 deaths in China alone (Arbyn et al., 2020). Over 95% of CC is attributed to human papillomavirus (HPV) infection. In November 2020, World Health Organization (WHO) launched a global strategy aimed at accelerating the elimination of CC as a public health concern. WHO set a goal to ensure that by the year 2030, the HPV vaccination coverage, screening coverage and treatment coverage for CC can, respectively, reach 90, 70 and 90% in all countries (Singh et al., 2023).

HPV is typically classified into high-risk and low-risk types. High-risk HPV types are oncogenic. Persistent HPV infection is identified as the primary cause in the development of cervical intraepithelial lesions, occurring in the majority of CC patients (Shamseddine et al., 2021). The viral oncoproteins E6 and E7 perform central roles on HPV-induced carcinogenic processes by affecting cell cycle and proliferation (Peng et al., 2024). Recently, integrating single-cell sequencing and spatial transcriptome revealed the mechanism of HPV-related CC carcinogenesis in terms of both the malignant transition of epithelial cells and the remodeling of the immune microenvironment (Guo et al., 2023). The study discovered critical genes that determined the cell fate between HPV infection and CC. Moreover, they observed imbalance of CD4+ T-cell subsets and exhaustion of CD8+ T cells in CC samples. To increase the rate of diagnosis and treatment for early CC, reduce CC mortality, and gradually establish a long-term mechanism to improve women’s health, the National Health Ministry of China launched a nationwide CC screening program for urban and rural female residents aged 35–64 years in 2009. From 2014, HPV genotyping was used as a pilot screening test. Nationwide, population-based studies showed the introduction of HPV testing could improve both the detection rate of CIN II-positive individuals and the efficiency of CC screening (Zhao et al., 2021; Bruni et al., 2022).

Due to the large population, the uneven levels of economic development and low vaccine coverage in China (Gao et al., 2023), the HPV infection rate, infection subtypes, and high-risk age range vary greatly in geographical distribution. Studies on HPV epidemiological features can provide a basis for the development of precise HPV prevention and treatment. Herein, we sought to perform a large-scale study from 2018 to 2023 to review local HPV epidemiology and its related cervical disease. The data included survey-based educational background, age, vaccination status, results of gynecological examination, HPV genotypes, cervical pathology, and treatment approaches. Different statistical models and bioinformatics algorithms were utilized to analyze the data. Our study aimed to present local epidemiological features of HPV and HPV-related cervical diseases. We hope to provide evidence to guide future HPV screening and vaccination strategies in China or other countries.

## 2 Materials and methods

### 2.1 Study population

A total of 149,559 urban and rural female residents aged 35–64 years who underwent the national HPV&CC screening program from 2018 to 2023 in Putian City were enrolled. The majority of the study population did not receive HPV vaccine. Exclusion criteria: cases with more than twice screening in the past 5 years, cases that have been diagnosed with CC or have undergone cervical surgery. Written informed consents were signed by all the participants.

### 2.2 Cervical cancer screening procedure

Local Maternity and Child Health Care Hospital is responsible for the organization and management of the screening program including investigating and advocating for the target population to undergo HPV screening. Strict quality control requirements were set throughout the entire process, including participant recruitment, information collection, sampling, sample transportation, laboratory testing, result verification and data security. Flow chart of the screening procedure was showed in Supplementary Figure S1.

### 2.3 HPV genotyping

HPV subtypes were detected by LumineX-200 (Luminex Company, United States), using Shanghai Toujing Life Science and Technology Company HPV genotyping kit (NO. 20173404697). HPV genotyping was performed strictly according to the manufacturer’s instructions. HPV DNA was extracted, and the 21 genotypes of HPV were detected. High-risk HPV types include subtypes 16, 18, 31, 33, 35, 39, 45, 51, 52, 53, 56, 58, 59, 66 and 68, while low-risk HPV types include subtypes 6, 11, 42, 43, 44 and CP8304.

### 2.4 Cytology and histopathological diagnosis

Specimens that were positive for high-risk HPV genotypes were directly used for ThinPrep cytologic test (TCT). The cytology diagnosis was classified as normal cervix, inflammation, atypical squamous cells of undetermined significance (ASC-US), and atypical squamous cells which cannot exclude HSIL (ASC-H) according to the Bethesda System (TBS). Patients with abnormal cytological results underwent colposcopy and histopathological examination. The results of pathological diagnosis were classified according to WHO 2014 classification as follows: Low-grade squamous intraepithelial lesion (LSIL) corresponding to cervical intraepithelial neoplasia grade (CIN) I, High-grade squamous intraepithelial lesion (HSIL) which describes CIN II or CIN III, and Invasive cervical carcinoma (ICC).

### 2.5 Statistical analysis

SPSS version 29.0.1.0 was used for the statistical analysis. The measurement data were presented as median (interquartile range/IQR). The enumeration data were described as frequency (percentage). The HPV prevalence between various disease types and the normal cervix was compared by the Chi-square test when the expected frequency was greater than 5. Otherwise, Fisher’s exact test was used. Bonferroni correction was implemented to control multiple comparison errors. Kruskal–Wallis H test was used to evaluate the differences in age among multiple groups. Chord diagrams were constructed to display HPV co-infection data. All statistical tests were two-sided, and *p* value less than 0.05 was considered statistically significant.

## 3 Results

### 3.1 Age and educational characteristics of the participants across all pathologic categories

**Figure 1:**
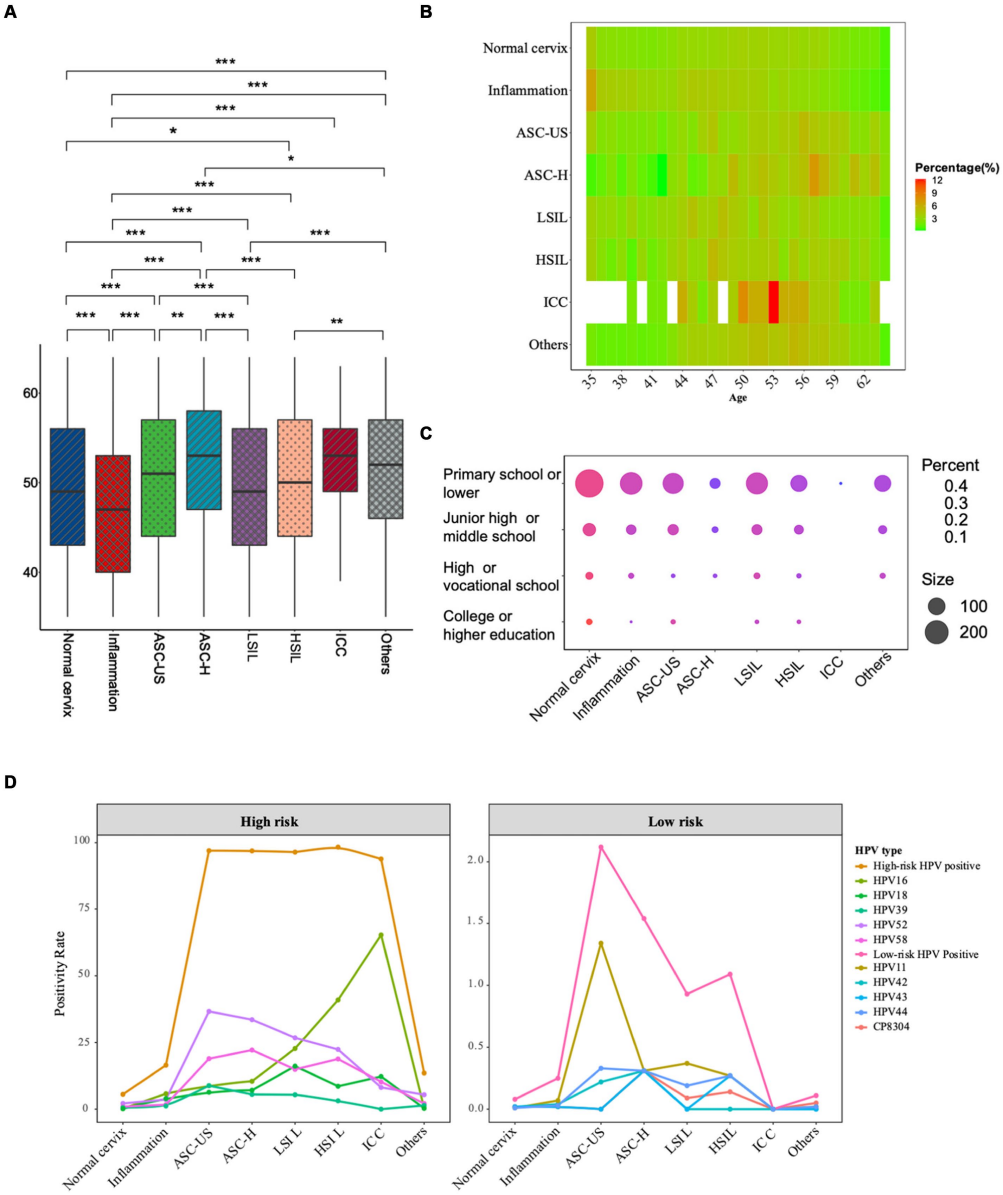
General characteristics of the participants across all pathologic categories. **(A)** Boxplot of age distribution according to pathology classification. The age distribution between various disease types and the normal cervix were compared by Kruskal–Wallis H test; **(B)** Heatmap of age distribution according to pathology classification. **(C)** Bubble plot of education levels (Y-axis) within different pathologic categories (X-axis). **(D)** The prevalence of top 5 prevalent high-risk and low-risk HPV types in different pathologic groups. Each color represents a different HPV type. (^***^The significance is at the *p* < 0.001 level. ^**^The significance is at the *p* < 0.01 level. ^*^The significance is at the *p* < 0.05 level).

A total of 149,559 females with a median age of 49 (IQR 43–56) years old were enrolled in the study. Among them, there were 129,523 cases of normal cervix (86.60%, 129,523/149,559), 10,667 cases of cervical inflammation (7.13%, 10,667/149,559), 898 cases of ASC-US (0.60%, 898/149,559), 325 cases of ASC-H (0.22%, 325/149,559), 1,073 cases of LSIL (0.72%, 1,073/149,559), 733 cases of HSIL (0.49%, 733/149,559), and 49 cases of ICC (0.03%, 49/149,559). Besides, 6,291 (4.2%, 6,291/149,559) cases showed other benign clinical features, which included cervical polyps, uterine fibroids and so on. As showed in Figures 1A,B, the median age was significantly different among pathologic categories (*p* < 0.05). The oldest median age was observed in ASC-H group with 54 years old, followed by ICC, ASC-US, HSIL and LSIL. The median age was 47 years in inflammation group, which was the youngest of all the groups.

A total of 1,233 participants provided educational experience anonymously. Considering the screened population mainly came from rural areas, the participants generally had low education levels. As showed in Figure 1C, individuals with primary or lower school education had the highest prevalence of all cervical diseases, including inflammation (82.86%, 87/105), ASC-US (81.72%, 219/268), ASC-H (79.31%, 46/58), LSIL (73.68%, 98/133), HSIL (81.25%, 52/64), ICC (100%, 1/1), and others (82.28%, 404/491). In contrast, those with college or higher education experiences generally had the lowest prevalence of all cervical diseases, with inflammation (0.95%, 1/105), ASC-US (1.87%, 5/268), ASC-H (0%, 0/58), LSIL (0.75%, 1/133), HSIL (1.56%, 1/64), ICC (0%, 0/1), and others (1.22%, 6/491). Individuals with a high school or vocational school and junior high school or middle school education exhibited intermediate prevalence rates in all cervical diseases.

### 3.2 HPV infection profile and abnormal pathological manifestations

The overall positive rate of HPV infection in 149,559 females was 8.60% (12,865/149,559). Among them, 8.58% (12,826/149,559) were infected with high-risk HPV, while 0.12% (179/149,559) were infected with low-risk HPV. The prevalence of HPV infections in different pathologic categories was presented in Table 1. The most prevalent high-risk HPV type in the screening population was HPV52, followed by HPV58, HPV16, HPV39, HPV51, HPV18, HPV68 and HPV33 in sequence. The positive rate of HPV infection was 5.60% in the normal cervix group. However, high infection rates were observed in ASC-US, ASC-H, LSIL, HSIL and ICC groups. In abnormal cervix groups including ASC-US, ASC-H, LSIL, HSIL and ICC, the positivity rates of all high-risk types were significantly higher than those in the normal cervix group (all *p* < 0.001). In inflammation cervix, the predominant prevalent HPV types were HPV16, HPV18 and HPV52. The predominant HPV types in ASC-US and ASC-H groups were HPV52 (36.64% in ASC-US, 33.54% in ASC-H), HPV58 (18.93% in ASC-US, 22.15% in ASC-H) and HPV16 (8.8% in ASC-US, 10.46% in ASC-H) with similar descending trend. HPV52, HPV16, HPV18, HPV58 and HPV51 were the top5 predominant types in LSIL. HPV16, HPV52, HPV58, HPV33 and HPV18 were the top5 predominant in HSIL patients. HPV16, HPV18, HPV58, HPV33 and HPV52 were predominant in ICC patients (see in Table 1). High-risk HPV types were found in normal cervix group with low prevalence. It should be noted that high-risk HPV types were more prevalent in abnormal cervix groups, with a particularly high prevalence of HPV16 in ICC patients of 65.31%. Low-risk HPV types were less common, with the highest prevalence in the ASC-US group, followed by the ASC-H group and the HSIL group.

**Table 1:** The HPV prevalence in different pathologic categories among 149,559 females.

HPV Type	Total	Normal cervix	Inflammation	ASC-US	ASC-H	LSIL	HSIL	ICC	Other
Number of cases	149,559	129,523	10,667	898	325	1,073	733	49	6,291
Positive	12,865 (8.60)	7,252 (5.60)	1,765 (16.55)^***^	873 (97.22)^***^	316 (97.23)^***^	1,037 (96.64)^***^	721 (98.36)^***^	46 (93.88)^***^	855 (13.59)^***^
Low-risk HPV Positive	179 (0.12)	103 (0.08)	27 (0.25)^***^	19 (2.12)^***^	5 (1.54)^***^	10 (0.93)^***^	8 (1.09)^***^	-	7 (0.11)
HPV6	30 (0.02)	12 (0.01)	8 (0.07)	2 (0.22)	1 (0.31)	3 (0.28)	2 (0.27)	-	2 (0.03)
HPV11	39 (0.03)	11 (0.01)	8 (0.07)	12 (1.34)	1 (0.31)	4 (0.37)	2 (0.27)	-	1 (0.02)
HPV42	34 (0.02)	27 (0.02)	4 (0.04)	2 (0.22)	1 (0.31)	-	-	-	-
HPV43	25 (0.02)	20 (0.02)	2 (0.02)	-	1 (0.31)	-	2 (0.27)	-	-
HPV44	30 (0.02)	18 (0.01)	3 (0.03)	3 (0.33)	1 (0.31)	2 (0.19)	2 (0.27)	-	1 (0.02)
CP8304	31 (0.02)	23 (0.02)	2 (0.02)	-	1 (0.31)	1 (0.09)	1 (0.14)	-	3 (0.05)
High-risk HPV Positive	12,826 (8.58)	7,227 (5.58)	1759 (16.49)^***^	871 (96.99)^***^	315 (96.92)^***^	1,035 (96.46)^***^	721 (98.36)^***^	46 (93.88)^***^	852 (13.54)^***^
HPV16	1,611 (1.08)	280 (0.22)	618 (5.79)	79 (8.80)	34 (10.46)	244 (22.74)	300 (40.93)	32 (65.31)	24 (0.38)
HPV18	976 (0.65)	211 (0.16)	423 (3.97)	57 (6.35)	23 (7.08)	173 (16.12)	63 (8.59)	6 (12.24)	20 (0.32)
HPV31	458 (0.31)	249 (0.19)	38 (0.36)	49 (5.46)	18 (5.54)	31 (2.89)	43 (5.87)	3 (6.12)	27 (0.43)
HPV33	714 (0.48)	405 (0.31)	58 (0.54)	44 (4.90)	25 (7.69)	66 (6.15)	69 (9.41)	4 (8.16)	43 (0.68)
HPV35	273 (0.18)	159 (0.12)	34 (0.32)	18 (2.00)	12 (3.69)	25 (2.33)	14 (1.91)	-	11 (0.17)
HPV39	1,115 (0.75)	722 (0.56)	127 (1.19)	79 (8.80)	18 (5.54)	58 (5.41)	23 (3.14)	-	88 (1.40)
HPV45	184 (0.12)	112 (0.09)	20 (0.19)	15 (1.67)	5 (1.54)	10 (0.93)	11 (1.50)	1 (2.04)	10 (0.16)
HPV51	997 (0.67)	616 (0.48)	95 (0.89)	68 (7.57)	22 (6.77)	99 (9.23)	24 (3.27)	1 (2.04)	72 (1.14)
HPV52	4,424 (2.96)	2,803 (2.16)	387 (3.63)	329 (36.64)	109 (33.54)	287 (26.75)	164 (22.37)	4 (8.16)	341 (5.42)
HPV53	183 (0.12)	118 (0.09)	12 (0.11)	17 (1.89)	10 (3.08)	13 (1.21)	7 (0.95)	-	6 (0.10)
HPV56	686 (0.46)	429 (0.33)	79 (0.74)	42 (4.68)	10 (3.08)	50 (4.66)	14 (1.91)	-	62 (0.99)
HPV58	2,102 (1.41)	1,243 (0.96)	180 (1.69)	170 (18.93)	72 (22.15)	160 (14.91)	138 (18.83)	5 (10.20)	134 (2.13)
HPV59	465 (0.31)	306 (0.24)	48 (0.45)	32 (3.56)	8 (2.46)	33 (3.08)	10 (1.36)	1 (2.04)	27 (0.43)
HPV66	502 (0.34)	303 (0.23)	51 (0.48)	37 (4.12)	10 (3.08)	51 (4.75)	9 (1.23)	1 (2.04)	40 (0.64)
HPV68	726 (0.49)	469 (0.36)	71 (0.67)	60 (6.68)	21 (6.46)	38 (3.54)	16 (2.18)	1 (2.04)	50 (0.79)

Compared by the Chi-square test. Normal cervix is served as control. ^***^The significance is at the *P* < 0.001 level. **The significance is at the *P* < 0.01 level. ^*^The significance is at the *P* < 0.05 level.

As depicted in Figure 1D and Table 1, the positive rate of HPV16 in inflammation, ASC-US, ASC-H, LSIL, HSIL and ICC were, respectively, 5.79, 8.80, 10.46%, 22.74, 40.93 and 65.31%, which had displayed an increasing trend with cancer progression. Conversely, the positive rate of HPV52 in ASC-US, ASC-H, LSIL, HSIL and ICC had showed a decreasing trend with cancer progression. Additionally, HPV18 prevalence in ICC was relatively lower than in LSIL (12.24% vs. 16.12%), whereas HPV33 exhibited increasing trend in HSIL and ICC. There were no low-risk HPV infections detected in patients with ICC.

### 3.3 HPV prevalence trend from 2018 to 2023

The HPV positive rate increased each year from 2018 to 2021 and stayed stable during 2022–2023 (see Table 2). The prevalence rate of HPV52 was the highest annually compared with other subtypes from 2018 to 2023. Moreover, in this six-year period, the prevalence of HPV52 in 2021 was the highest with the positive rate of 3.76%. The prevalence of high-risk HPV, including HPV39 and 58, slightly increased during this period. The prevalence of all low-risk HPV types was generally lower compared to high-risk types. The screening of low-risk HPV types began in 2021, so there was no data available for low-risk HPV types of 2018 and 2020.

**Table 2:** HPV prevalence trend from 2018 to 2023.

HPV	2018	2019	2020	2021	2022	2023
Number of cases	2,000	15,000	31,095	36,231	30,114	35,119
Positive cases	36 (1.80)	1,104 (7.36)	2,427 (7.81)	3,370 (9.30)	2,779 (9.23)	3,149 (8.97)
Low-risk HPV Positive	–	53 (0.35)	–	27 (0.07)	95 (0.32)	4 (0.01)
HPV6	–	20 (0.13)	–	4 (0.01)	3 (0.01)	3 (0.01)
HPV11	–	28 (0.19)	–	6 (0.02)	5 (0.02)	–
HPV42	–	-	–	4 (0.01)	30 (0.10)	–
HPV43	–	3 (0.02)	–	5 (0.01)	16 (0.05)	1 (<0.01)
HPV44	–	2 (0.01)	–	11 (0.03)	17 (0.06)	–
CP8304	–	–	–	–	31 (0.10)	–
High-risk HPV Positive	36 (1.80)	1,083 (7.22)	2,427 (7.81)	3,365 (9.29)	2,766 (9.19)	3,149 (8.97)
HPV16	18 (0.90)	175 (1.17)	298 (0.96)	408 (1.13)	340 (1.13)	372 (1.06)
HPV18	3 (0.15)	98 (0.65)	202 (0.65)	258 (0.71)	208 (0.69)	207 (0.59)
HPV31	3 (0.15)	61 (0.41)	71 (0.23)	84 (0.23)	111 (0.37)	128 (0.36)
HPV33	2 (0.10)	59 (0.39)	223 (0.72)	164 (0.45)	150 (0.50)	116 (0.33)
HPV35	–	35 (0.23)	36 (0.12)	46 (0.13)	75 (0.25)	81 (0.23)
HPV39	–	92 (0.61)	185 (0.59)	243 (0.67)	290 (0.96)	305 (0.87)
HPV45	–	26 (0.17)	21 (0.07)	35 (0.10)	43 (0.14)	59 (0.17)
HPV51	3 (0.15)	87 (0.58)	156 (0.50)	188 (0.52)	264 (0.88)	299 (0.85)
HPV52	4 (0.20)	290 (1.93)	897 (2.88)	1,364 (3.76)	866 (2.88)	1,003 (2.86)
HPV53	–	22 (0.15)	48 (0.15)	62 (0.17)	49 (0.16)	2 (0.01)
HPV56	1 (0.05)	31 (0.21)	120 (0.39)	149 (0.41)	153 (0.51)	232 (0.66)
HPV58	3 (0.15)	148 (0.99)	430 (1.38)	577 (1.59)	453 (1.50)	491 (1.40)
HPV59	1 (0.05)	20 (0.13)	64 (0.21)	109 (0.30)	138 (0.46)	133 (0.38)
HPV66	–	42 (0.28)	73 (0.23)	99 (0.27)	117 (0.39)	171 (0.49)
HPV68	2 (0.10)	68 (0.45)	100 (0.32)	144 (0.40)	188 (0.62)	224 (0.64)

### 3.4 Single and multiple HPV infections

**Figure 2:**
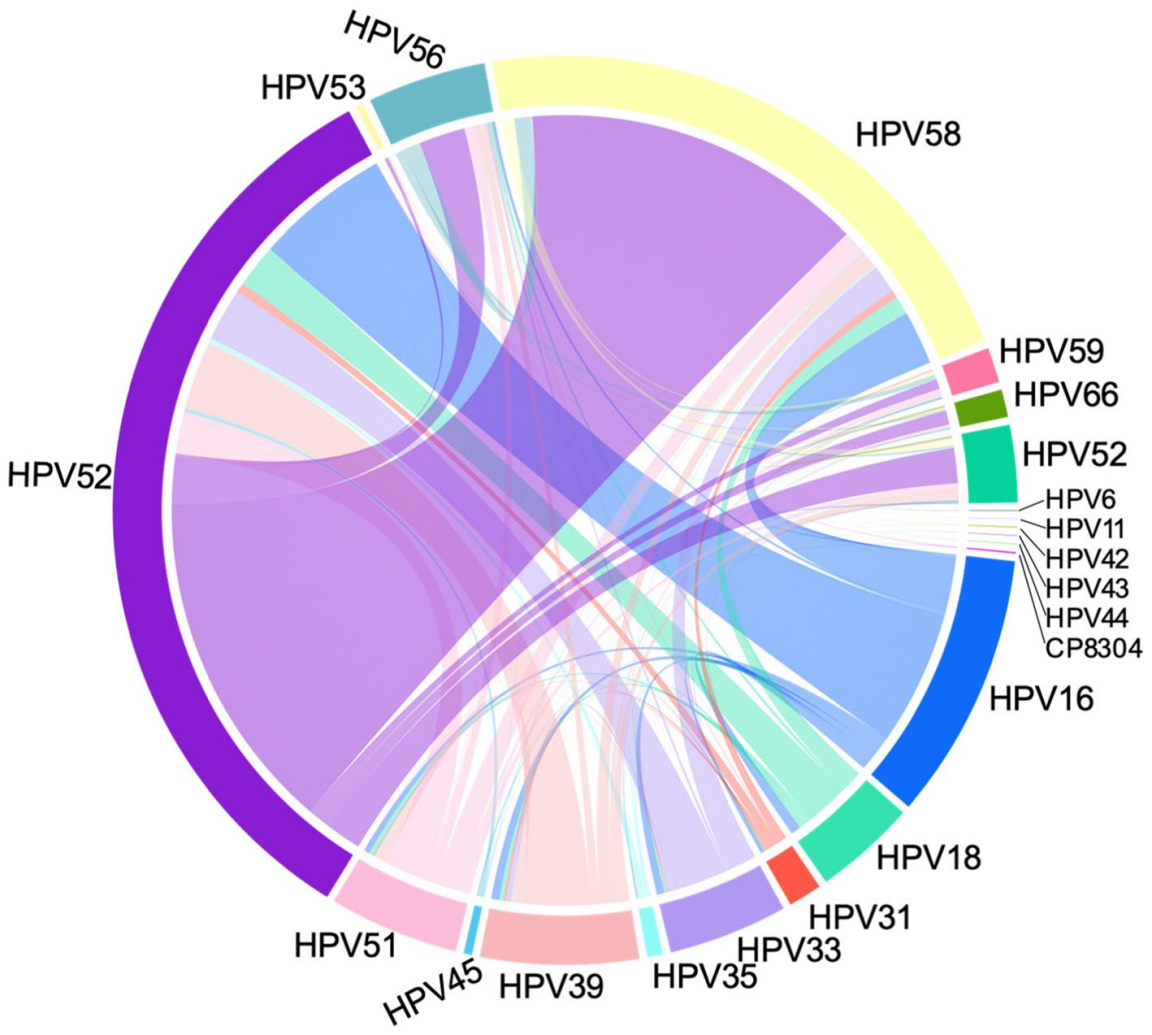
A chord diagram depicting the co-infections status of distinct HPV types.

Among the 12,865 HPV-infected females, 82.59% were infected with a single HPV type, and 99.65% single infection were high-risk HPV subtypes. 17.41% females suffered from multiple HPV infections. Among them, 81.70% had dual HPV subtype infections, 15.13% had triple HPV subtype infections, and 3.17% had quadruple or more HPV subtype infections. The prevalence of single and multiple infections was significantly different in inflammation, ASC-US, LSIL and HSIL patients compared to normal cervix group (all *p* < 0.001). The chord plot in Figure 2 clearly displayed the co-infections status of distinct HPV types. The predominant co-infection combinations of HPV subtypes were HPV52 plus HPV58, HPV16 plus HPV52 and HPV39 plus HPV52. The plot showed that HPV52, HPV58 and HPV16 were the most frequently combined subtypes. As the age increased, the proportion of multiple HPV infections became more pronounced (Supplementary Figure S2). In patients over 55 years old, the percentage of multiple HPV infections was significantly higher than single infection, particularly in the 55–64 age group (see Table 3).

**Table 3:** Single and multiple HPV infection within different pathologic groups.

HPV infection	Total	Normal cervix	Inflammation	ASC-US	ASC-H	LSIL	HSIL	ICC	Other
Number of cases	12,865 (100.00)	7,252 (56.37)	1,765 (13.72)	873 (6.79)	316 (2.46)	1,037 (8.06)	721 (5.60)	46 (0.36)	855 (6.65)
Single infection	10,625 (82.59)	6,176 (85.16)	1,375 (77.90)^***^	675 (77.32)^***^	253 (80.06)	789 (76.08)^***^	564 (78.22)^***^	35 (76.09)	758 (88.65)
Single low-risk	37 (0.35)	23 (0.37)	6 (0.44)	2 (0.30)	1 (0.40)	2 (0.25)	–	–	3 (0.40)
Single high-risk	10,588 (99.65)	6,153 (99.63)	1,369 (99.56)	673 (99.70)	252 (99.60)	787 (99.75)	564 (100.00)	35 (100.00)	755 (99.60)
Multiple infection	2,240 (17.41)	1,076 (14.84)	390 (22.10)^***^	198 (22.68)^***^	63 (19.94)	248 (23.92)^***^	157 (21.78)^***^	11 (23.91)	97 (11.35)
Double infection	1830 (81.70)	904 (84.01)	294 (75.38)	161 (81.31)	47 (74.60)	199 (80.24)	129 (82.17)	9 (81.82)	87 (89.69)
Triple infection	339 (15.13)	144 (13.38)	82 (21.03)	30 (15.15)	10 (15.87)	38 (15.32)	23 (14.65)	2 (18.18)	10 (10.31)
Quadruple or more	71 (3.17)	28 (2.60)	14 (3.59)	7 (3.54)	6 (9.52)	11 (4.44)	5 (3.18)	–	–

Compared by the Chi-square test. Normal cervix is served as control. ^***^The significance is at the *P* < 0.001 level. **The significance is at the *P* < 0.01 level. *The significance is at the *P* < 0.05 level.

### 3.5 Age-specific HPV prevalence and type distribution

**Figure 3:**
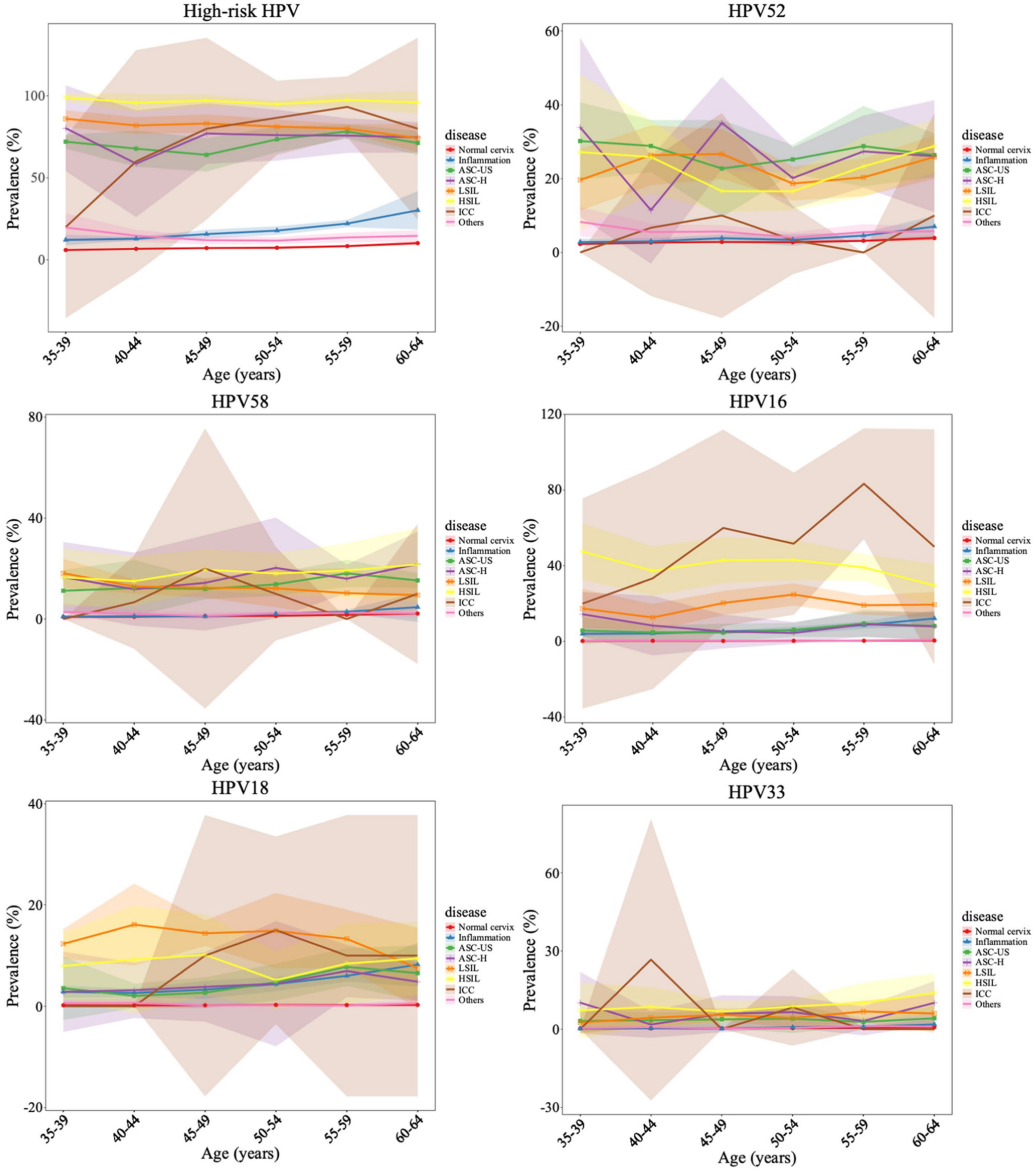
Age-specific HPV prevalence and its correlations with cervical pathology change. The points represent the HPV prevalence in each age group, with the 95% confidence intervals are indicated with colored shading.

The relationship between age and HPV infection was showed in Figure 3 and Supplementary Figure S3. The prevalence of all HPV subtypes exhibited upward trend with increasing age. The lowest prevalence was observed in cases aged 35–39. It slightly increased among cases aged 40–49 but increased significantly in those aged 50–64. The highest prevalence of HPV was observed in cases aged 60–64. The peak prevalence of high-risk HPV in ICC patients was observed in cases aged 55–59. Regarding the most prevalent type HPV52, it had two prevalence peaks in ASC-H females aged 35–39 and 45–49. The peak HPV16 prevalence was observed in ICC group in cases aged 45–49 and 55–59. HPV18 infection was the most popular type in LSIL group among all ages. In addition, the peak prevalence of HPV33 was observed in younger individuals aged 40–44 who developed ICC. The characteristics of other subtypes were displayed in Supplementary Figure S4.

## 4 Discussion

According to Lancet Global Health 2022, only 9% of women in low- and middle-income countries had received HPV screening ever in lifetime. In low- and middle-income countries, the burden of HPV-related diseases is the heaviest (Hu et al., 2023). In 2020, the CC mortality rate in all 78 low- and middle-income countries was estimated at 13.2 cases per 100,000 women (Canfell et al., 2020). In our study, 149,559 females with a median age of 49 years (IQR 43–56) were involved. We observed 86.60% of the screened population had a normal cervix, while 7.13% had inflammation, 0.60% had ASC-US, 0.22% had ASC-H, 0.72% had LSIL, 0.49% had HSIL, and 0.03% had ICC. The latest U.S. guideline recommended a combination of HPV testing with cytology for CC screening in women aged 30 to 65 (Liu et al., 2021). The National Health Service of the United Kingdom implemented the cytology-based CC screening service to all females aged 25 to 64, which has observed a decline in CC incidence (Choi et al., 2023). Our findings indicated that the highest median age was observed in ASC-H group with 54 years old, followed by ICC, ASC-US, HSIL and LSIL. The median age was 47 years in inflammation group, which was the youngest of all the groups.

Numerous clinical trials have demonstrated that HPV-based screening is more effective than cytology (Chan et al., 2020; Zhang et al., 2021). HPV prevalence varies significantly across different countries and regions (Rogua et al., 2023). Different HPV prevalence rates have been reported, including 26.8% in the United States (Dunne et al., 2007), 11.3% in Northern America (de Sanjosé et al., 2007), 2–12% in Europe (de Vuyst et al., 2009), and 25% in Japan (Sasagawa et al., 2016). In our study, the HPV prevalence among urban and rural female residents was 8.60%, and most of them were high-risk subtypes. The prevalence was lower than that of outpatient population, which ranged from 18.42 to 31.94% in 37 Chinese cities (Wang et al., 2015). In our screened population, HPV52 was the most common subtype, followed by HPV58, HPV16, HPV39, HPV51, HPV18, HPV68 and HPV33 in sequence. The data from 198,111 routine cervical screenings in Guangzhou city revealed distinct epidemiological characteristics of HPV subtypes, with a notably lower ranking of HPV18 (Yang et al., 2023). HPV prevalence increased with the severity of cervical lesions (Forman et al., 2012). Our observations showed that the prevalence of HPV infection was 5.60% in normal cervix group. In groups with abnormal cervical pathology, the HPV infection rate exceeded 90%, suggesting a strong correlation between HPV infection and cervical disease outcomes.

Our results showed that in China, the most prevalent HPV types in inflamed cervix were HPV16, HPV18 and HPV52. The infection caused irritation and activated inflammasome to cervix. Studies showed that E6/E7 from HPV16 and HPV18 upregulated IL-6 expression, with IL-6 as one of the main pro-inflammatory cytokines involved (Artaza-Irigaray et al., 2019). In ASC-US and ASC-H, the predominant HPV types in order of prevalence are HPV52, HPV58, and HPV16. In LSIL, HPV52, HPV16, HPV18, HPV58 and HPV51 were the top5 predominant types. HPV16, HPV52, HPV58, HPV33 and HPV18 were the top5 predominant in HSIL. HPV16, HPV18, HPV58, HPV33 and HPV52 were predominant in ICC patients. In a 67,742 women study (Lu et al., 2021), the prevalence rates of HPV16 and HPV18 were significantly higher in ICC. It was reported that HPV 16 and HPV 18 infections exceeded 70% in cervical cancer patients (Soerjomataram and Bray, 2021). Our results showed that the prevalence rates of HPV16 in inflammation, ASC-US, ASC-H, LSIL, HSIL and ICC were, respectively, 5.79, 8.80, 10.46%, 22.74, 40.93 and 65.31%, which indicated an increasing trend correlating with cancer progression, suggesting its critical role in cancer progression. Conversely, the prevalence of HPV52 in ASC-US, ASC-H, LSIL, HSIL and ICC showed a decreasing trend with cancer progression. HPV33 infection were increased in HSIL and ICC. Previous reports suggested HPV33 as the second frequent type in prostate cancer (Ahmed et al., 2023). Consistent with the results of a large-scale study in Chengdu, the top five genotypes with HPV16 and HPV18, in co-infection included HPV33 (Wei et al., 2023). Therefore, a special attention should be paid to HPV33 infection. The screening strategies in China have been continually optimized based on local epidemiological characteristics. Considering HPV-positive women with normal cytology remained at increased risk of CIN III (Polman et al., 2017), in addition to HPV16 and 18, whether HPV33-infected females needed to be referred for colposcopy to avoid missed diagnosis is worth investigating.

Previous studies reported two age peaks in HPV prevalence among the general population in China: one at 15–24 years and another at 35–49 years (Song et al., 2020). Another 10-year survey demonstrated the first age peak below 25 years and the second above 56 years (Wei et al., 2022). Our findings demonstrated that the infection rates of all HPV subtypes exhibited an upward trend with increasing age. For high-risk HPV, the peak age range in ICC cases was 55–59 years. HPV52 had two prevalence peaks in ASC-H females aged 35–39 and 45–49. The peak HPV16 prevalence was observed in ICC group in cases aged 45–49 and 55–59. HPV18 infection was the most popular type in LSIL group among all ages. In addition, the peak prevalence of HPV33 was observed in younger individuals aged 40–44 who developed ICC. The age-specific peak observed could be associated with immunologic function caused by hormonal changes. At perimenopausal period, the hormone secretion is reduced, the imbalance of vaginal microbiota provides conditions for cervix infection (Laniewski et al., 2020). Usually, increased risk of ICC is observed 15–30 years after HPV infection (Gilham et al., 2023). Since HPV33 has the highest prevalence in females aged 40–44 who developed ICC. Recent study showed that cervical carcinogenesis risk was associated with HPV33 E6 and E7 genetic variations (Yan et al., 2023). Special attention should be paid to HPV33 screening and vaccination strategy.

Among the 12,865 HPV-infected females, 17.41% females suffered from multiple HPV infections. The prevalence of single infection was 82.59%, higher than 65.9% (Tao et al., 2022a) and 57.2% (Tao et al., 2022b) reported in Shanghai. The prevalence of single and multiple infections was significantly different in inflammation, ASC-US, LSIL and HSIL patients compared to normal cervix group. However, the effect of multiple-subtype infections on the risk of CIN remained controversial, and some multiple-subtype infection patterns were benign while others were strongly associated with progression to CC (Depuydt et al., 2016). Compared with single infection, sometimes multiple HPV infections had a lower risk of HSIL, which might be related to its higher clearance rate (Ni et al., 2023). HPV52 plus HPV58, HPV16 plus HPV52, and HPV39 plus HPV52 were the TOP 3 combinations of double co-infection. The prevalence of multiple HPV infections in Beijing was reported to exhibit a bimodal age distribution with first peak below 25 years, and a second peak above 55 years, whereas single HPV infections exhibited one peak aged 35–44 years (Li et al., 2019). Our study showed multiple HPV infections accounted for a larger proportion in females above 55 years old, with a peak at 55–64 years.

The annual positive rate increased from 2018 to 2021 and stayed stable during 2022 to 2023. HPV52 was the most prevalent subtype consistently. The prevalence of high-risk HPV types including HPV39 and 58 slightly increased during this period. Our survey showed that individuals with primary school education or lower had the highest prevalence in all cervical diseases. In contrast, those with college or higher education generally had the lowest positive rates. Chinese government started to implement nationwide free HPV vaccination project for school-aged girls in 2022. A cross-sectional study in Beijing pointed out that lower educational levels were the primary reason for vaccine refusal (Liu et al., 2020). Policies and measures, such as community health education and public health planning, are necessary to enhance awareness of HPV screening and improve the vaccination rate. Large and long-term retrospective study is needed to strengthen the management of HPV-positive females.

## 5 Conclusion

HPV-based screening with cytology was an effective screening method. HPV16, HPV18, HPV58, HPV33 and HPV52 are the top5 infection subtypes in ICC patients. Special attention should be paid to HPV33 infection especially in females aged 40–44 years old who had a higher risk of ICC.

## Data availability statement

The raw data supporting the conclusions of this article will be made available by the authors, without undue reservation.

## Ethics statement

The studies involving humans were approved by The Medical Ethics Committee of the Affiliated Hospital of Putian University. The studies were conducted in accordance with the local legislation and institutional requirements. The participants provided their written informed consent to participate in this study.

## Author contributions

KL: Writing – review & editing, Writing – original draft. QH: Writing – original draft. YF: Writing – review & editing. HT: Writing – review & editing, Validation, Supervision. HL: Writing – review & editing, Methodology, Data curation. JH: Writing – review & editing, Methodology. YH: Writing – review & editing, Resources, Methodology. MH: Writing – review & editing, Methodology. MC: Writing – review & editing, Supervision, Project administration, Funding acquisition.
