# Supplementary material for: Cervical HPV infection and related diseases among 149,559 women in Fujian: an epidemiological study from 2018 to 2023
Source: Front Microbiol. 2024 Jun 19;15:1418218. doi: 10.3389/fmicb.2024.1418218 (PMC11220154; doi:10.3389/fmicb.2024.1418218)
Supplement: Supplementary file 1 [file Data_Sheet_1.docx]

**Cervical HPV infection and related diseases among 149,559 women in Fujian:**

**an epidemiological study from 2018 to 2023**

**Supplementary materials**


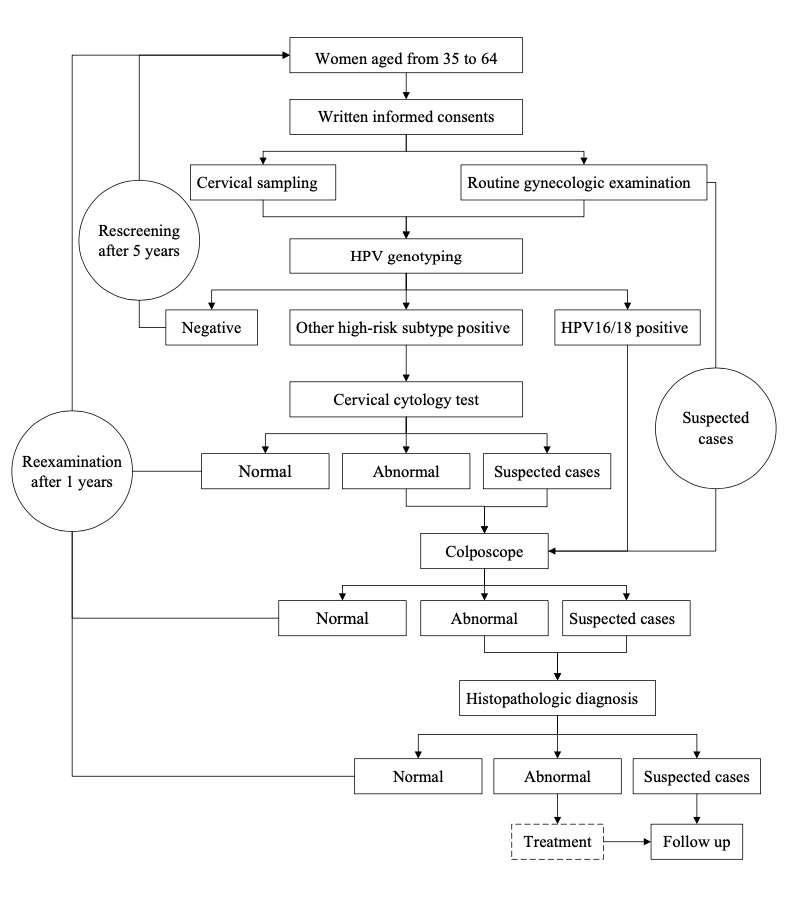


Figure S1. Flow chart of the screening procedure


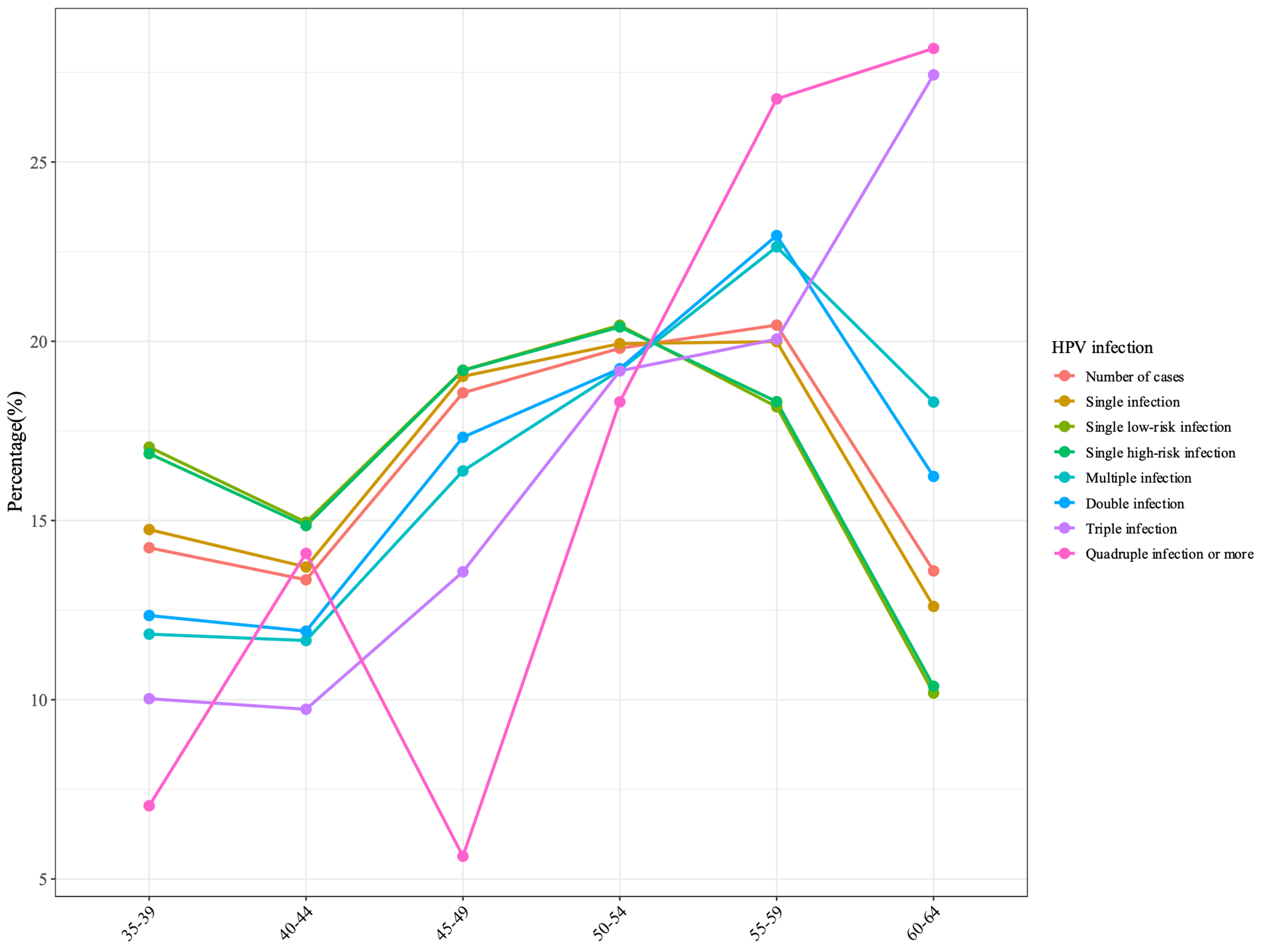


Figure S2. HPV co-infection status in different age groups


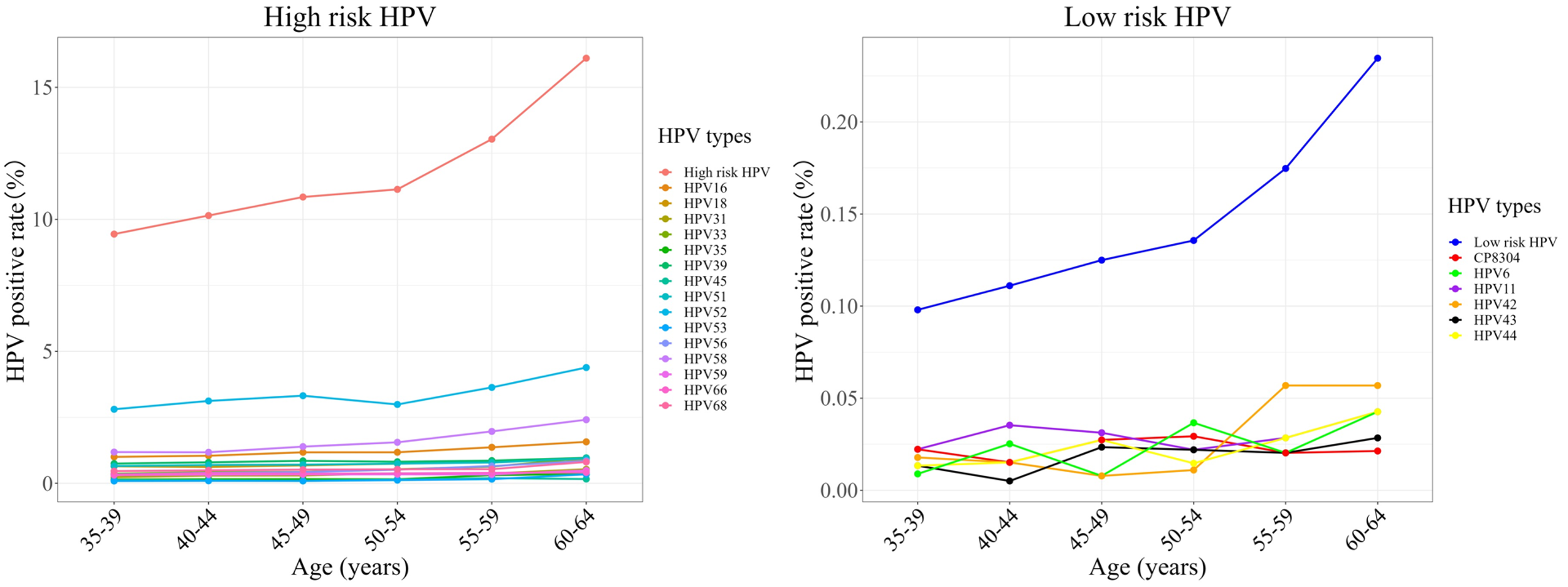


Figure S3. HPV positive rate across different age groups


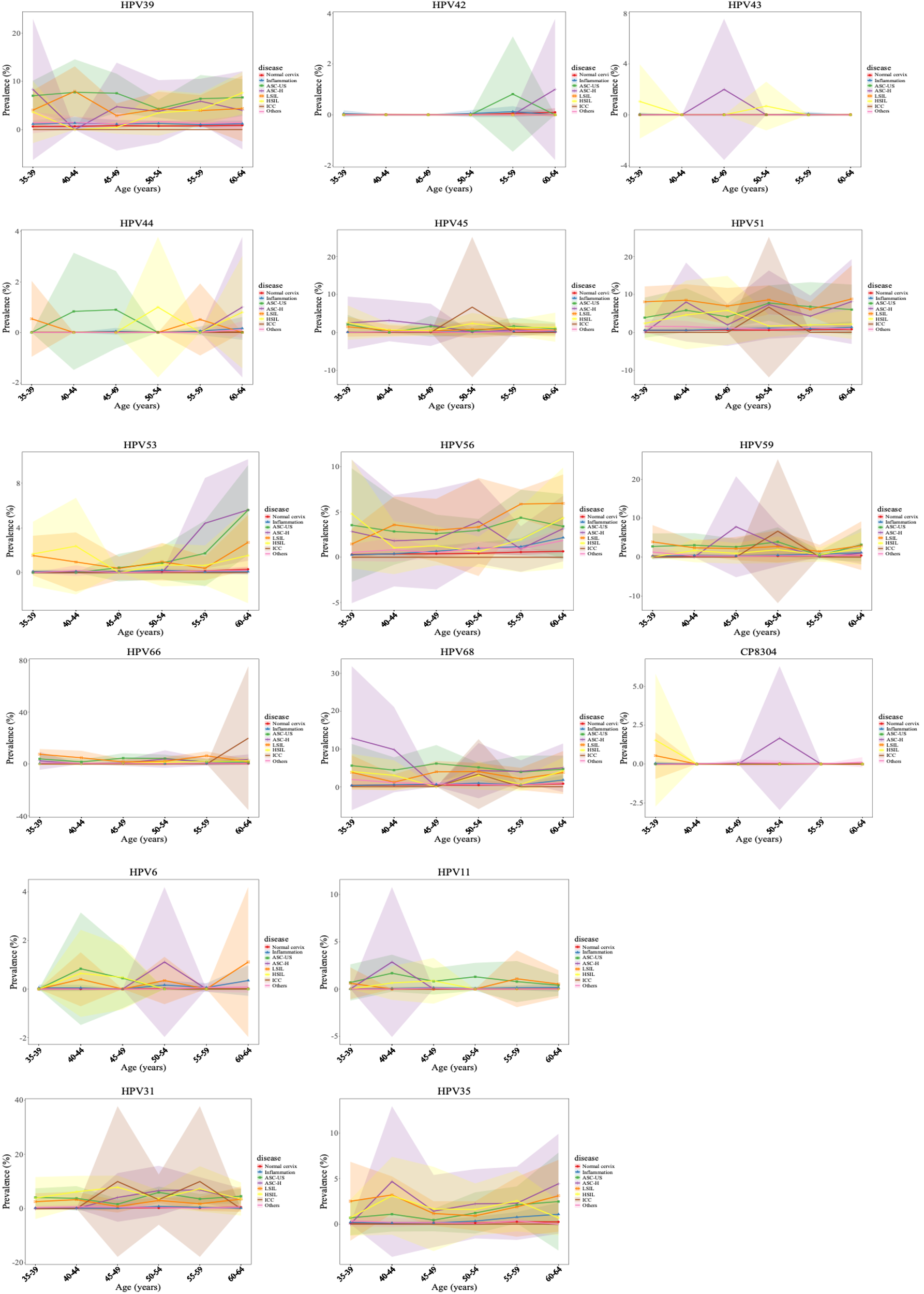


Figure S4. Age-specific HPV prevalence and its correlations with cervical pathology changes（All subtypes）
